# Internet Addiction and Its Associated Factors Among African High School and University Students: Systematic Review and Meta-Analysis

**DOI:** 10.3389/fpsyg.2022.847274

**Published:** 2022-03-21

**Authors:** Edgeit Abebe Zewde, Tadesse Tolossa, Sofonyas Abebaw Tiruneh, Melkalem Mamuye Azanaw, Getachew Yideg Yitbarek, Fitalew Tadele Admasu, Gashaw Walle Ayehu, Tadeg Jemere Amare, Endeshaw Chekol Abebe, Zelalem Tilahun Muche, Tigabnesh Assfaw Fentie, Melkamu Aderajew Zemene, Metages Damite Melaku

**Affiliations:** ^1^Department of Biomedical Sciences, College of Health Sciences, Debre Tabor University, Debre Tabor, Ethiopia; ^2^Department of Public Health, Institute of Health Sciences, Wollega University, Nekemte, Ethiopia; ^3^Department of Public Health, College of Health Sciences, Debre Tabor University, Debre Tabor, Ethiopia; ^4^Department of Medicine, College of Health Sciences, Debre Tabor University, Debre Tabor, Ethiopia

**Keywords:** internet addiction, adolescent, Africa, problematic internet use, systematic review and meta-analysis

## Abstract

**Introduction:**

Internet addiction is characterized by excessive and uncontrolled use of the internet affecting everyday life. Adolescents are the primary risk group for internet addiction. Data on internet addiction is lacking in Africa. Thus, this review aimed to determine the pooled prevalence of internet addiction and its associated factors among high school and university students in Africa.

**Methods:**

A comprehensive literature search was conducted using electronic databases (PubMed/MEDLINE, Web of science, Hinari, and Google scholar) to locate potential studies. Heterogeneity between studies was checked using Cochrane Q test statistics and I^2^ test statistics and small-study effects were checked using Egger's statistical test at a 5% significance level. A sensitivity analysis was performed. A random-effects model was employed to estimate the pooled prevalence and associated factors of internet addiction among students. The primary outcome of measure of this review was the prevalence of internet addiction and the secondary outcome of measures are the factors associated with internet addiction.

**Results:**

A total of 5,562 studies were identified among the five databases. Of these, 28 studies from 10 countries with 14,946 high school and university students were included in this review. The overall pooled prevalence of internet addiction among the students was 34.53% [95% Confidence Interval (CI): 26.83, 42.23, I^2^ = 99.20%]. Male sex [Pooled Odds Ratio (POR) = 1.92, 95% CI:1.43, 2.57 I^2^ = 0.00], urban residence (POR = 2.32, 95% CI:1.19, 4.53, I^2^ = 59.39%), and duration of daily internet use for more than 4 h (POR = 2.25, 95% CI:1.20, 4.21, I^2^ = 0.00%, were significantly associated with internet addiction among adolescents.

**Conclusion:**

Almost one-third of university and high school students in Africa are addicted to the internet. Male students, those from urban areas, and those who use the internet for more than 4 h per day have higher odds of internet addiction. Thus, we recommend that health planners and policymakers pay attention to the use of the internet and internet addiction in Africa.

## Introduction

Internet and smartphone use has increased worldwide over the recent decades and has become a critical part of modern-day life [World Health Organization (WHO), 2014]. Statistics show that as of January 2021, the global population using the internet has grown to almost 4.6 billion (Kemp, 2021). In Africa, internet use increased from 120 million in 2014 to 270 million in 2019 (Okae and Gyasi, 2013).

Appropriate internet use is important in communications, research, socialization, entertainment, and many other benefits. However, internet use also has negative impacts, in which internet overuse has been associated with addiction and mental health issues (Chao et al., 2020).

Internet addiction is a heterogeneous problem including different online activities including online gaming, pornography, social networking, and online shopping (Nissen, 2006). Since 2013 Internet gaming disorder is included in the 11th edition of the International Classification of Diseases (ICD-11) as a clinically significant problem and in the classification of mental and behavioral disorders of the American Psychiatric Association (DSM-5) as a condition for further study (World Health Organization, 2018). Despite its heterogeneity Internet addiction does not yet exist as a diagnosis or specific disorder in either ICD-11 or DSM-5 (Musetti et al., 2016).

Internet addiction is characterized by excessive and uncontrolled use of the internet affecting everyday life (Young, 1998). The disorder is mainly associated with other psychological conditions including attention deficit hyperactivity disorder and depression (Silvia Kratzer1, 2007). The global prevalence is estimated to be 6% ranging from 2.6 % in western Europe to 10.25% in the Middle East (Cheng and Li, 2014).

Adolescents are the primary risk group for internet addiction (Öztürk and Özmen, 2016). Developmental changes in the brain during adolescence especially in cognition, stress, and motivation make these age groups more vulnerable to addictive behaviors (Casey et al., 2005; Hammond et al., 2014).

Internet addiction in adolescence can have multiple negative impacts including cognitive problems (Park et al., 2011), loneliness (Hasmujaj, 2016), poor family and interpersonal relationship problems (Seo et al., 2009; Hou et al., 2019) poor school performance (Javaeed et al., 2020; Hamza, 2021), lower self-esteem (Yedemie, 2021), lack of selfcare (Tran et al., 2017), depression (Ha et al., 2007; Guo et al., 2012; Iskender, 2014; Ansar et al., 2020), anxiety stress (Silvia Kratzer1, 2007; ElSalhy et al., 2019; Saikia et al., 2019; Boudabous et al., 2020), and obesity (Bozkurt et al., 2018; Arafa et al., 2020; Citlik-Saritas et al., 2020).

Literature shows that different factors predispose adolescents to internet addiction: including urban residence, presence of internet at home (Effat et al., 2019; Abd El-Mawgood et al., 2021), gender (Akpunne et al., 2020; Nyaga, 2020), spending more hours on the internet (Abd et al., 2017; Fantaw, 2021; Kapus et al., 2021), using internet for entertainment, pornography and online gaming (Seo et al., 2009; Asrese and Muche, 2020; Zenebe et al., 2020), and substance use (Tran et al., 2017; Zenebe et al., 2020; Kapus et al., 2021).

Even though internet addiction is becoming a global concern its assessment and criteria to define internet addicts is still a challenge (Byun et al., 2009). Despite its shortcomings, the Internet Addiction Test (IAT) is one of the most widely used tests in assessing internet addiction (Sela et al., 2021). The test has gained international acceptance and has shown to have reliability and consistency (Moon et al., 2018). Different terminologies have been used to describe internet addiction problems in different literature. “Internet addiction,” “Problematic internet use” and “pathological use of internet” are the most commonly used terminologies (Pau, 2019).

Studies have been conducted to assess the prevalence of internet addiction in different regions with inconsistent and inconclusive findings. There is no pooled systematic review and meta-analysis which assesses the prevalence of internet addiction among adolescents in Africa. Therefore, this review aims to determine the pooled prevalence of internet addiction and its associated factors among college and university students in Africa. The findings from this study can be used by health planners and policymakers to curve the prevalence of internet addiction among African students.

## Methods

### Study Setting and Search Strategy

Searches were conducted on PubMed/MEDLINE, Web of science, Hinari, and Google scholar. Additionally, unpublished works were reviewed from research centers and library sources. Systematic searches of all electronic databases were conducted from October to November 2021. Pre-identified search terms were used to allow a comprehensive search strategy that included all the relevant studies. Pre-identified search terms such as “Internet addiction” OR “internet addiction disorder” OR “problematic internet use” OR “cyber addict” OR “smartphone addiction” OR “social media addiction” OR “media addiction” were used.

### Eligibility of Criteria

We used the CoCoPop (Condition, Context, and Population) approach for prevalence studies to declare the inclusion and exclusion criteria.

### Inclusion and Exclusion Criteria

Studies reporting the prevalence of internet addiction in university and high school students in any of the African countries using Young's Internet addiction test (YIAT) were included. Additionally, full-text articles written in English were included in the review. Studies conducted on postgraduate students; studies that used tools other than YIAT were excluded.

### Measurement of the Outcome Variable

The primary measure of the outcome of this review was the prevalence of internet addiction according to young's internet addiction Test. The IAT questionnaire was used as a screening tool to examine the level of Internet addiction. The tool was developed by Young and colleagues in 1996. It contains 20 items to examine symptoms of IA based on a 5-point Likert scale ranging from 0 to 5 (0 = not applicable, 1 = rarely, 2 = occasionally, 3 = frequently, 4 = often, 5 = always). According to the tool, the severity of IA is scored as follows: 20–49 points is “average Internet users”; 50–79 points are “possible problematic Internet users”; and 80– 100 points is “severe Internet addict.” The cutoff points for all studies included in this review were standardized in this review, a score below 50 was classified as “normal internet use” and a score of 50 and above was classified as “internet addiction” (Young, 1998).

The second outcome was the factors associated with internet addiction, which were determined using the odds ratio (OR) and calculated based on binary outcomes from the included primary studies.

### Study Selection and Data Collection

All the studies reviewed through different electronic databases were combined, exported, and managed using Endnote version X7.2 (Thomson Reuters, Philadelphia, PA, USA) software. All duplicate studies were removed and full-text studies were downloaded using Endnote software and manually. The eligibility of each study was assessed independently by two reviewers (EA. and SA.). Subsequently, studies were screened and excluded based on their titles and abstracts. Full-text articles or reports were assessed in the remaining articles. The eligibility of the studies was evaluated based on the predetermined inclusion and exclusion criteria. Differences in the results of the two reviewers narrowed through discussion and other reviewer members (TT and GY).

### Assessment of Quality of Individual Studies

Hoy quality assessment tool was used to assess the quality of the studies. It has nine questions. Based on the score of the quality assessment tool, the lowest score had the minimum risk of bias. Overall scores range from (0–3), (4–6), and (7–9), which are declared low, moderate, and high risk of bias respectively (Kirthi et al., 2021). Three reviewers independently assessed the studies (EAZ, TT, and SAT). Disagreements between them were resolved by another review team (GWA and ECA).

### Data Extraction and Management

Three reviewers (EAZ, SAT, and MMA) independently extracted the data using a standardized data extraction checklist on a Microsoft Excel spreadsheet. The discrepancies between the two authors were managed by discussion and by other reviewers (TT, GYY, TAF, and FTA). For each study, authors, year of publication, region, study design, sample size, the prevalence of internet addiction with standard error, and determinant factors, with effect size and their standard error were extracted.

### Statistical Analysis

After extraction, data were exported to STATA/MP version 16.0 software for analysis. The pooled prevalence of internet addiction and its associated factors were analyzed by the random-effects model using Der Simonian-Laird model weight (DerSimonian and Laird, 1986). Statistically, significant heterogeneity was assessed using the Cochrane Q-test and I^2^ statistics (Higgins et al., 2019). To minimize the variance of estimated points between primary studies, a subgroup analysis was carried out on the regions, economies, and target groups. A sensitivity analysis was conducted to determine the influence of single studies on pooled estimates. Univariate meta-regression was conducted using the year of publication and the mean age in the study using a random-effects model. Publication bias (small study effect) was checked graphically using a funnel plot and Egger's statistical test (Egger et al., 1997). Statistically significant Egger's test (*P*-value < 0.05) indicates the presence of a small study effect, and is handled by non-parametric trim and fill analysis using the random-effects model (Duval and Tweedie, 2000).

## Results

### Study Selection and Identification

**Figure 1:**
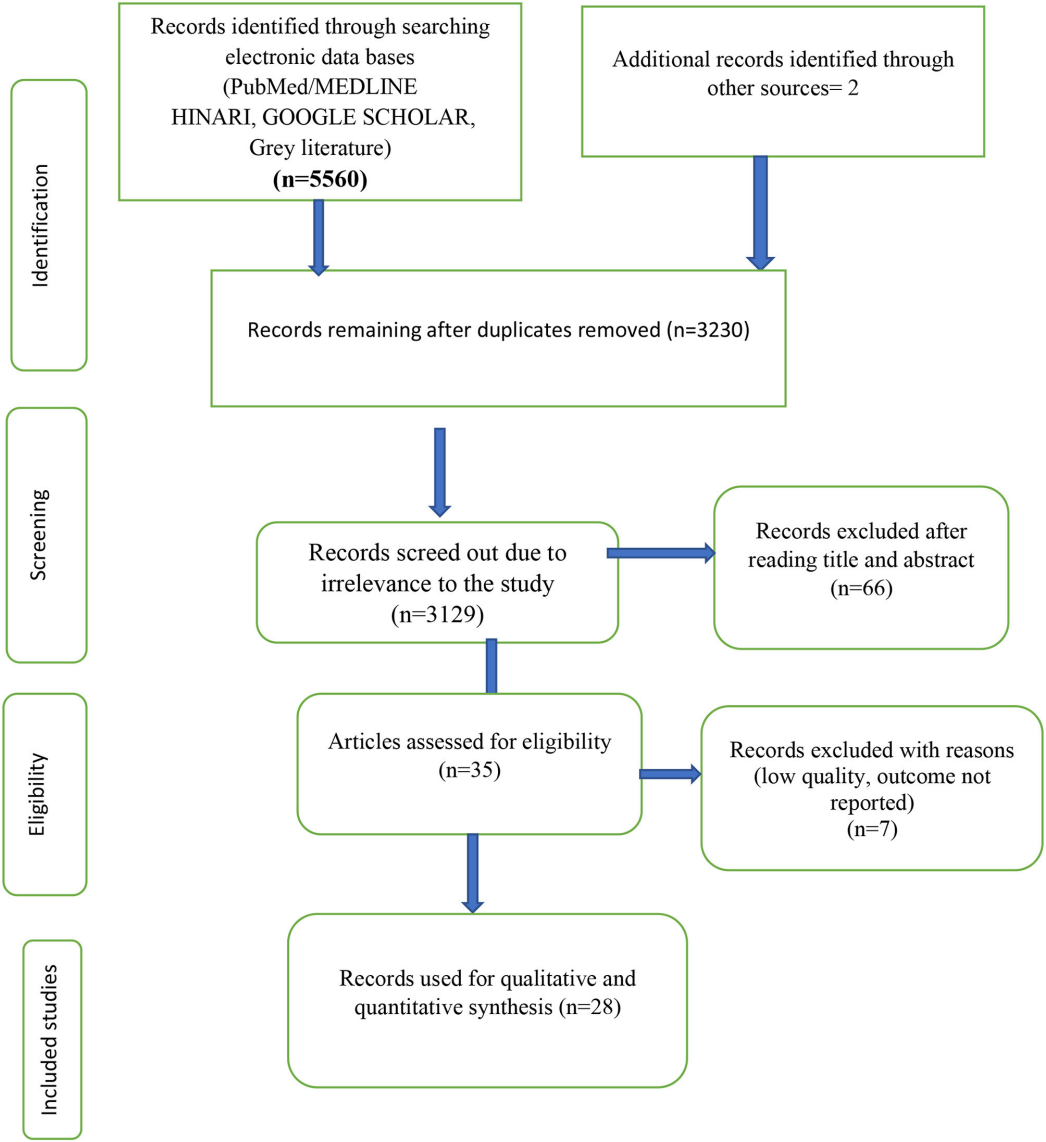
Prisma flow diagram of article selection for systematic review and meta-analysis on the prevalence of internet addiction and its associated factors among students in Africa.

Of the 5,562 papers searched from different databases, 2,332 duplicates were removed, and 3,129 were removed because of irrelevance to the study; again 66 papers were removed by reading the title and abstract. Finally, out of the remaining 35 papers, 7 were excluded due to low quality, the outcome not reported, and tests other than YIAT. Finally, twenty-eight papers were used for the meta-analysis (Figure 1).

### Characteristics of Included Studies

All the included studies were cross-sectional studies. A total of 14,946 high school and university students were included in the review. The mean age was 19.44 ± 2.80 years. The minimum and the maximum ages were 14.15 and 24.4 years, respectively. The minimum prevalence (7.7 %) of internet addiction was reported in Nigeria (Ilesanmi et al., 2021). The maximum prevalence (88.3%) was reported in a study conducted in Egypt (Ebrahim Essa and Elsherif, 2020) (Table 1).

**Table 1:** Characteristics of included studies in the study of review on prevalence of internet addiction and its associated factors among adolescents in Africa.

**S/N**	**References**	**Country**	**Region**	**Sample**	**Prevalence**	**Quality**
				**size**	**of IA**	**score**
1	Reda et al., 2012	Egypt	North Africa	501	22.9	1
2	Kamal and Mosallem, 2013	Egypt	North Africa	605	20.8	1
3	Chérif et al., 2015	Tunisia	North Africa	587	18.05	1
4	Alhajjar, 2014	Egypt	North Africa	1,656	52.3	2
5	Missaoui and Brahim, 2015	Tunisia	North Africa	982	11.6	3
6	Chinatu, 2015	Nigeria	West Africa	200	13.5	1
7	Moges, 2017	Ethiopia	East Africa	369	26	1
8	Goorah and Fuzoolla, 2018	Mauritius	South Africa	372	67.2	1
9	Asrese and Muche, 2020	Ethiopia	East Africa	812	35.2	1
10	Shaheen and Farahat, 2016	Egypt	North Africa	396	48.5	1
11	Ogachi et al., 2019	Kenya	East Africa	400	16.6	3
12	Arafa et al., 2020	Egypt	North Africa	828	52.8	1
13	Effat et al., 2019	Egypt	North Africa	588	35.2	1
14	Mohamed and Bernouss, 2020	Morocco	North Africa	305	72.7	1
15	Yedemie, 2021	Ethiopia	East Africa	359	25.3	1
16	Iluku-Ayoola et al., 2020	Nigeria	West Africa	147	44.2	1
17	Ebrahim Essa and Elsherif, 2020	Egypt	North Africa	273	88.3	1
18	Zenebe et al., 2020	Ethiopia	East Africa	548	29.3	1
19	Boudabous et al., 2020	Tunisia	North Africa	120	21.2	1
20	Mboya et al., 2020	Tanzania	East Africa	500	31	1
21	Fantaw, 2021	Ethiopia	East Africa	304	28.2	3
22	Amoah et al., 2020	Ghana	West Africa	122	9.8	3
23	Salama, 2020	Egypt	North Africa	608	47.5	1
24	Omoyemiju and Popoola, 2021	Nigeria	West Africa	1,448	44.5	1
25	Ilesanmi et al., 2021	Nigeria	West Africa	376	7.7	1
26	Abd El-Mawgood et al., 2021	Egypt	North Africa	400	25	1
27	Study and Mengistu, 2021	Ethiopia	East Africa	846	19.4	1
28	Hamza, 2021	Sudan	East Africa	321	66.6	1

### The Pooled Prevalence of Internet Addiction Among Youth

**Figure 2:**
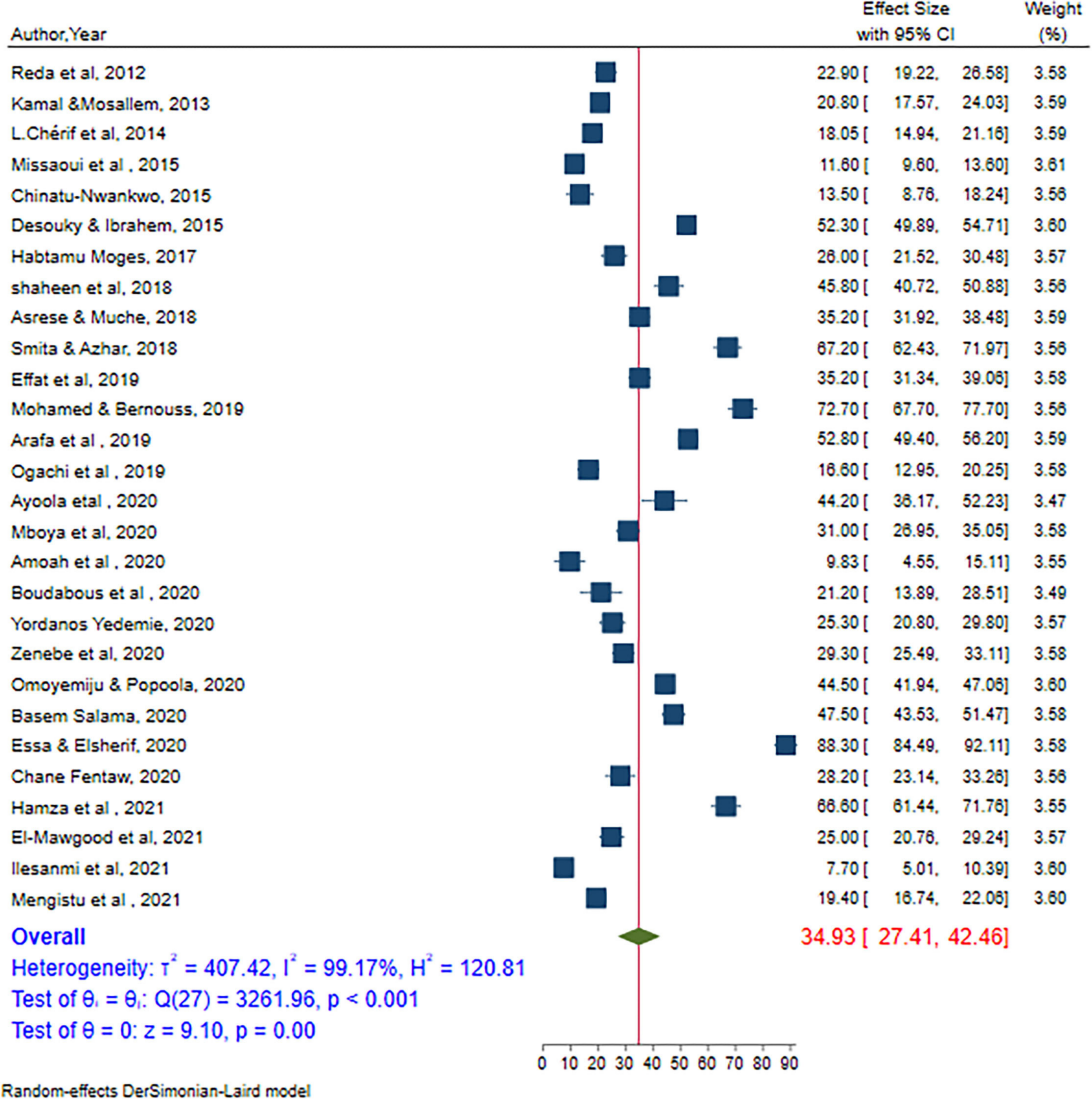
The pooled prevalence of internet addiction among high school and university students in Africa.

In the random effects model, the pooled prevalence of internet addiction was 34.93% (95% CI = 27.41–42.45). Significant heterogeneity was observed among studies (I^2^ = 99.17, *P*-value < 0.001) (Figure 2). There was no publication bias as evidenced by Egger's test bias (β = 0.12, *P*-value = 0.07) (Supplementary Figure 1).

### Handling Heterogeneity

Significant heterogeneity was observed from the random-effects model. To handle this heterogeneity; sensitivity analysis, subgroup analysis, and meta-regression analysis were performed. In sensitivity analysis, there were no studies that excessively influence the pooled prevalence of internet addiction. Subgroup analysis was performed based on the region and target population, Based on the region, the highest prevalence of internet addiction was observed in a study conducted in Mauritius 63% (Goorah and Fuzoolla, 2018), and the lowest prevalence was observed in the West African region 28.37%. Based on the target population, the highest prevalence of internet addiction was observed in studies conducted among university students as compared with studies conducted among high school students, 36.93 and 28.87% respectively (Table 2).

**Table 2:** Sub-group analysis for internet addiction and its associated factors among students in Africa.

**Variables**		**Included studies**	**Sample size**	**Prevalence (95%CI)**	**Heterogeneity (I^**2**^, *P*-value)**
By region	North Africa	12	7,453	39.03 (25.54–52.52)	99.48%, ≤ 0.001
	East Africa	9	4,459	30.76 (22.63–38.90)	97.47%, <0.001
	West Africa	5	2,293	23.87 (5.33–42.41)	99.13%, <0.001
	South Africa	1	372	67.20 (62.43–71.97)	0.00
By target group	High school	8	4,584	28.87 (15.97–41.76)	99.25%, <0.001
	University	20	10617	36.93 (28.10–45.76)	98.99%, <0.001
By socioeconomic status	Low income	7	3,559	32.78 (22.83–42.74)	97.79%, <0.001
	Low middle income	19	10,646	33.45 (23.61–43.69)	98.15%, <0.001
	Upper middle income	1	372	67.20 (62.43–71.97)	0.00%

### Meta-Regression

Meta-regression analysis was computed to evaluate underlying sources of heterogeneity using mean age, study quality, and year of publication. No significant association was observed between internet addiction and the above-described variables (Table 3).

**Table 3:** Univariate meta-regression analysis result for the prevalence of internet addiction among African college and university students.

**Study level variables**	**Adjusted R^**2**^**	**Standard error**	**Coefficients (95% CI)**
Mean age	8.33	1.53	1.64 (−1.36–4.64)
Year of publication	0.00	1.52	1.74 (−1.24–4.73)
Study quality	0.89	5.91	−10.95 (−22.15–0.64)

*CI, Confidence Interval*.

### Internet Addiction and Factors Associated

**Figure 3:**
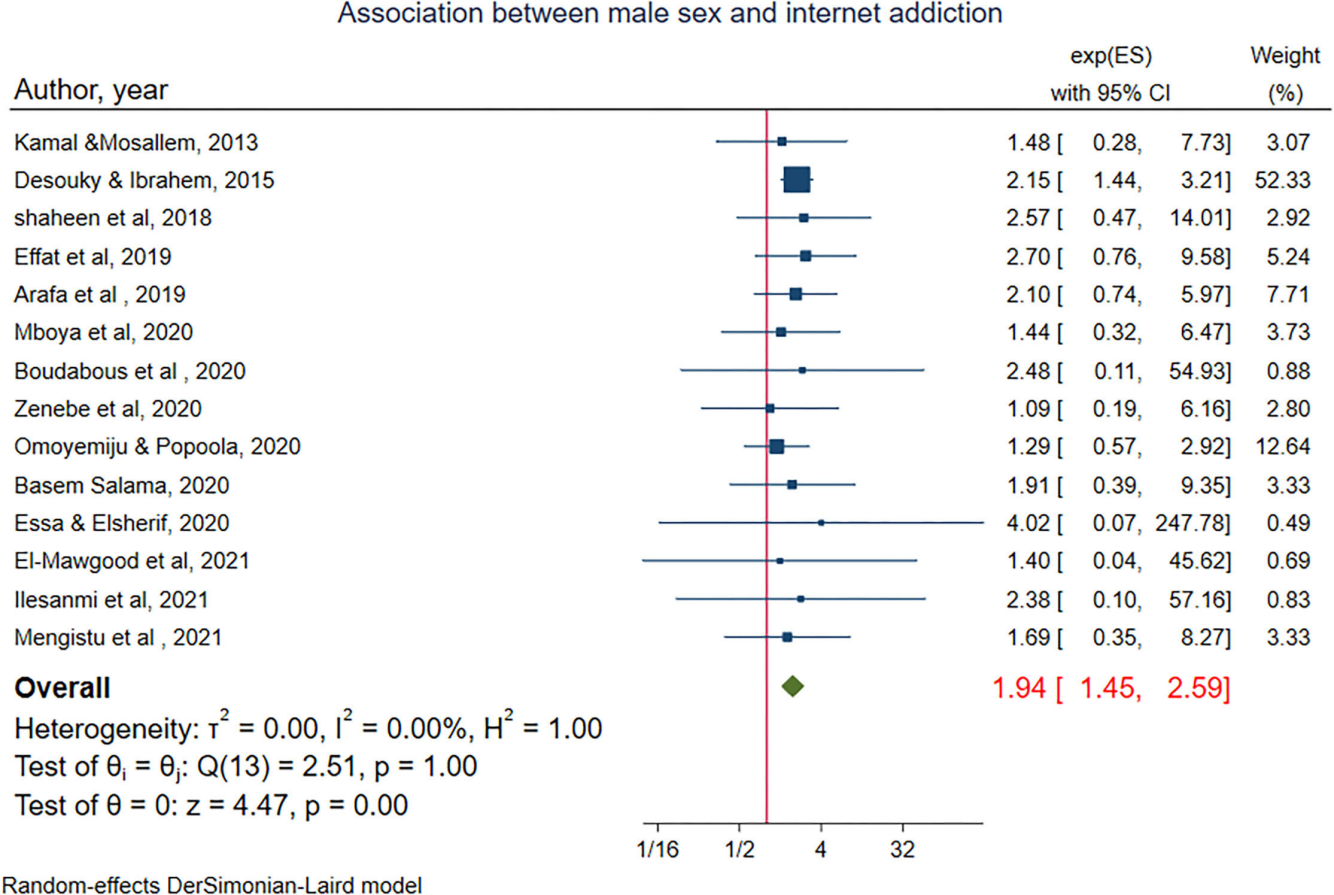
The pooled association between sex and internet addiction.

To examine the association of internet addiction with sex, 14 studies were included (Kamal and Mosallem, 2013; Alhajjar, 2014; Shaheen and Farahat, 2016; Effat et al., 2019; Arafa et al., 2020; Boudabous et al., 2020; Ebrahim Essa and Elsherif, 2020; Mboya et al., 2020; Salama, 2020; Zenebe et al., 2020; Abd El-Mawgood et al., 2021; Ilesanmi et al., 2021; Omoyemiju and Popoola, 2021; Study and Mengistu, 2021). Among these, 10 studies reported a significantly higher odds of internet addiction among male adolescents (Alhajjar, 2014; Shaheen and Farahat, 2016; Effat et al., 2019; Arafa et al., 2020; Boudabous et al., 2020; Ebrahim Essa and Elsherif, 2020; Salama, 2020; Ilesanmi et al., 2021; Omoyemiju and Popoola, 2021; Study and Mengistu, 2021). The pooled estimate showed a significant association between sex and internet addiction. Male adolescents are 94% more likely to have internet addiction as compared with their counterparts [POR = 1.94, 95% CI:(1.45–2.59)]. In the random-effects model, no heterogeneity was observed among studies, (I^2^ = 00.00%, *P*-value 1.0) (Figure 3). There was no small study effect as evidenced by Egger's test (*P*-value = 0.71). Additionally, there was no single study that excessively influences the estimate of the effect size (Supplementary Figure 2).

**Figure 4:**
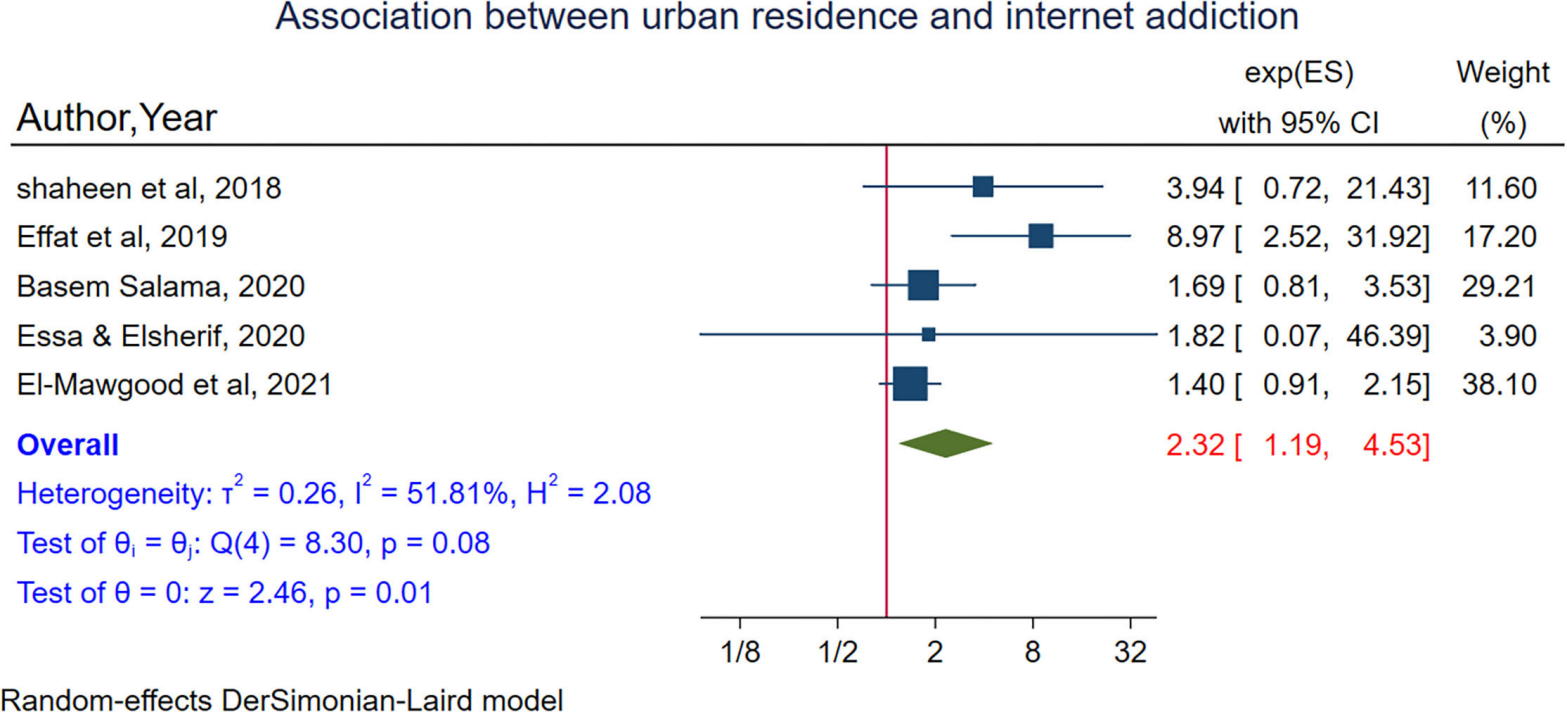
The pooled association between residence and internet addiction.

In a meta-analysis of five studies (Shaheen and Farahat, 2016; Effat et al., 2019; Ebrahim Essa and Elsherif, 2020; Salama, 2020; Abd El-Mawgood et al., 2021), adolescents that reside in urban areas are 2 times more likely to have internet addiction as compared to those who reside in rural areas (POR = 2.32, 95% CI:1.19–4.53). Moderate heterogeneity was observed in the random-effects model (I^2^ = 59.39%, *P*-value = 0.08) (Figure 4). Egger's test showed no small study effect (*P*-value = 0.20) and in sensitivity analysis, no study influences the estimates (Supplementary Figure 3).

**Figure 5:**
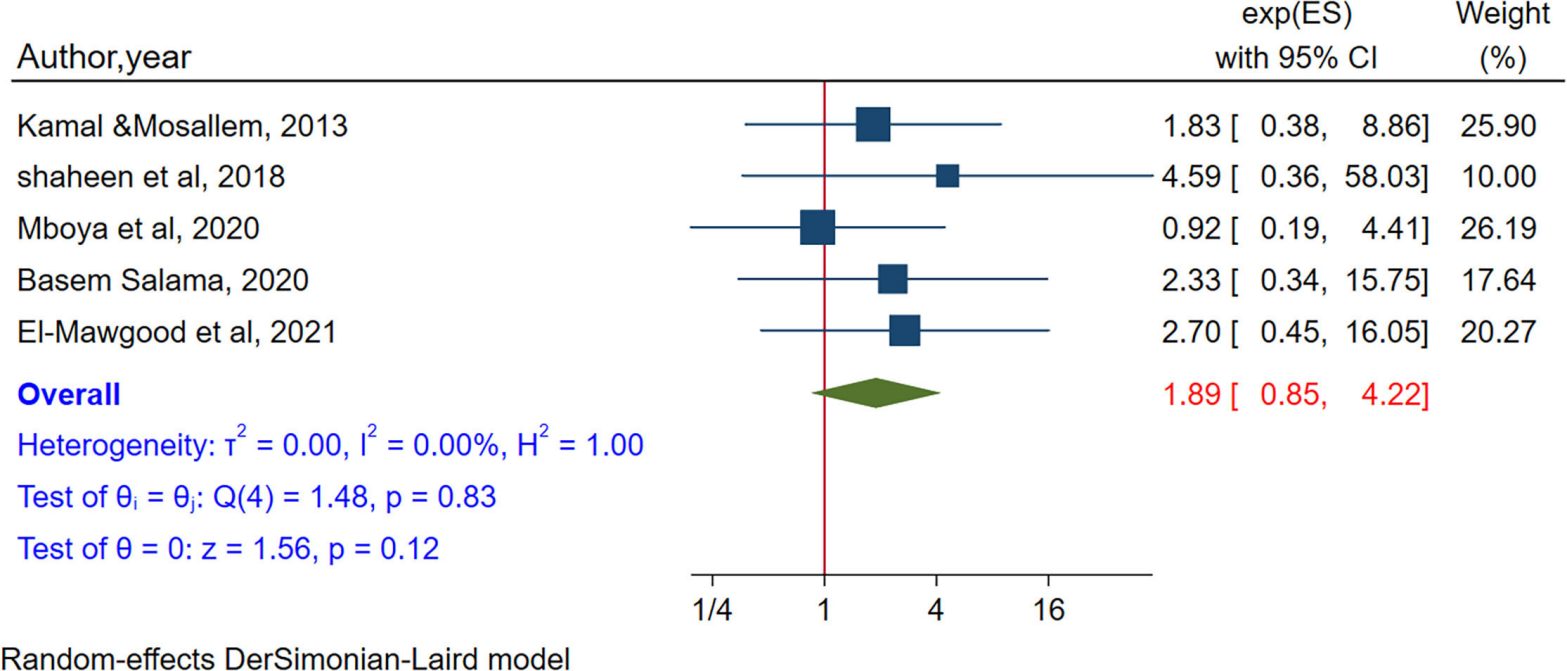
The pooled association between availability of internet at home and internet addiction.

To see the association between availability of internet at home, five studies were included (Kamal and Mosallem, 2013; Shaheen and Farahat, 2016; Mboya et al., 2020; Salama, 2020; Abd El-Mawgood et al., 2021). Among these studies, four (Kamal and Mosallem, 2013; Shaheen and Farahat, 2016; Salama, 2020; Abd El-Mawgood et al., 2021) studies reported significantly higher odds of internet addiction among adolescents who have internet at home. In the pooled estimate, no significant association was observed between the availability of internet at home and internet addiction (POR = 1.89, 95% CI:0.85–4.22). No heterogeneity was observed between studies (I^2^ = 0.00%, *P*-value = 0.83) (Figure 5), and no small study effect was evidenced by Egger's test (*P*-value = 0.33). In sensitivity analysis, no single study influences the estimates (Supplementary Figure 4).

**Figure 6:**
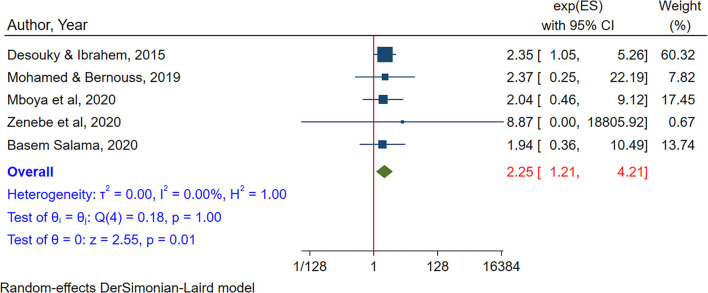
The pooled association between duration of internet use and internet addiction.

In a meta-analysis of four studies (Alhajjar, 2014; Mboya et al., 2020; Mohamed and Bernouss, 2020; Salama, 2020), duration of internet use was found to be significantly associated with internet addiction. Adolescents who use the internet more than 4 h per day were two times more likely to have internet addiction as compared to their counterparts (POR = 2.23, 95% CI:1.19– 4.18). There was no heterogeneity between studies in the random effects model (I^2^ = 0.00%, *P*-value = 1) (Figure 6). There was no small study effect as evidenced by Egger's test (*P*-value = 0.88). And by sensitivity analysis, there was no study that excessively influences the pooled association between duration of internet use and internet addiction (Supplementary Figure 5).

**Figure 7:**
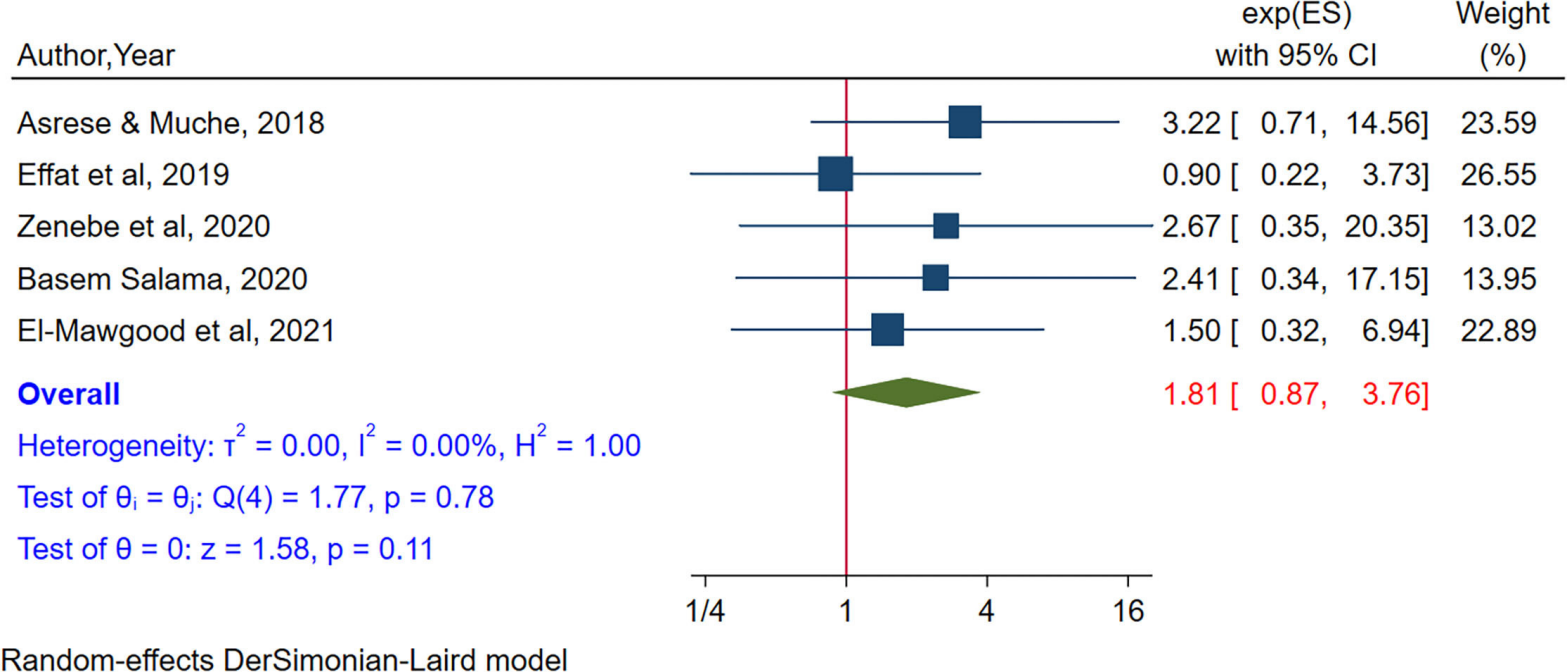
The pooled association between gaming and internet addiction.

Five studies (Effat et al., 2019; Asrese and Muche, 2020; Salama, 2020; Zenebe et al., 2020; Abd El-Mawgood et al., 2021) were included to assess the pooled association between gaming and internet addiction. The pooled result showed no significant association between gaming and internet addiction (POR = 1.80, 95% CI:0.87–3.76). No heterogeneity was observed from the random effects model (I^2^ = 0.00%, *P*-value = 0.78) (Figure 7). Egger's test showed no small study effect (*P*-value = 0.49), and in the sensitivity analysis, there was no study that influences the estimates (Supplementary Figure 6).

**Figure 8:**
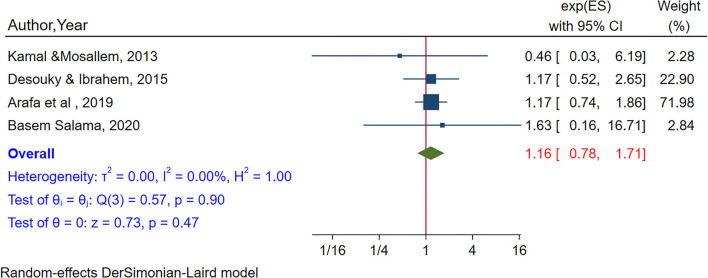
The pooled association between smoking and internet addiction.

To estimate the association between smoking and internet addiction, four studies were included (Kamal and Mosallem, 2013; Alhajjar, 2014; Arafa et al., 2020; Salama, 2020). No significant association was seen between smoking and internet addiction (POR = 1.16, 95% CI:0.78–1.71). No heterogeneity was observed from the random effects model (I^2^ = 0.00%, *P*-value = 0.92) (Figure 8). There was no small study effect by Egger's test (*P*-value = 0.88) and in sensitivity analysis, there was no single study that influences the pooled estimates (Supplementary Figure 7).

**Figure 9:**
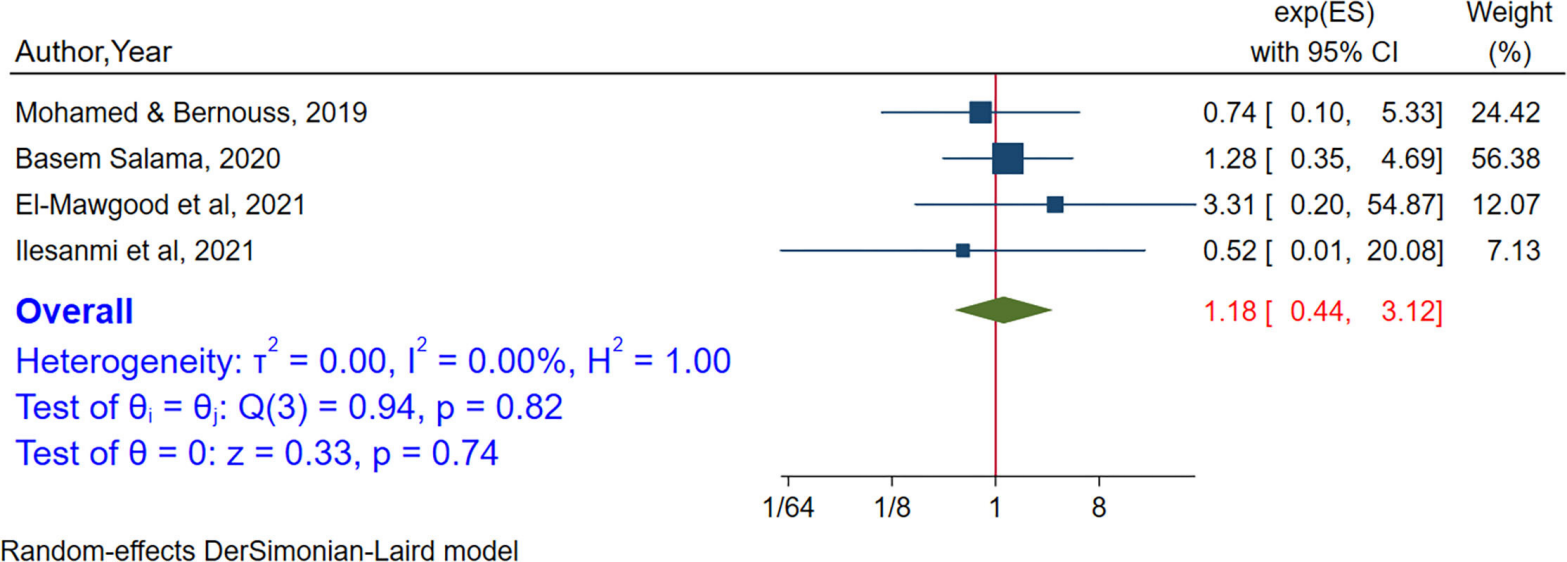
The pooled association between mothers' education and internet addiction.

**Figure 10:**
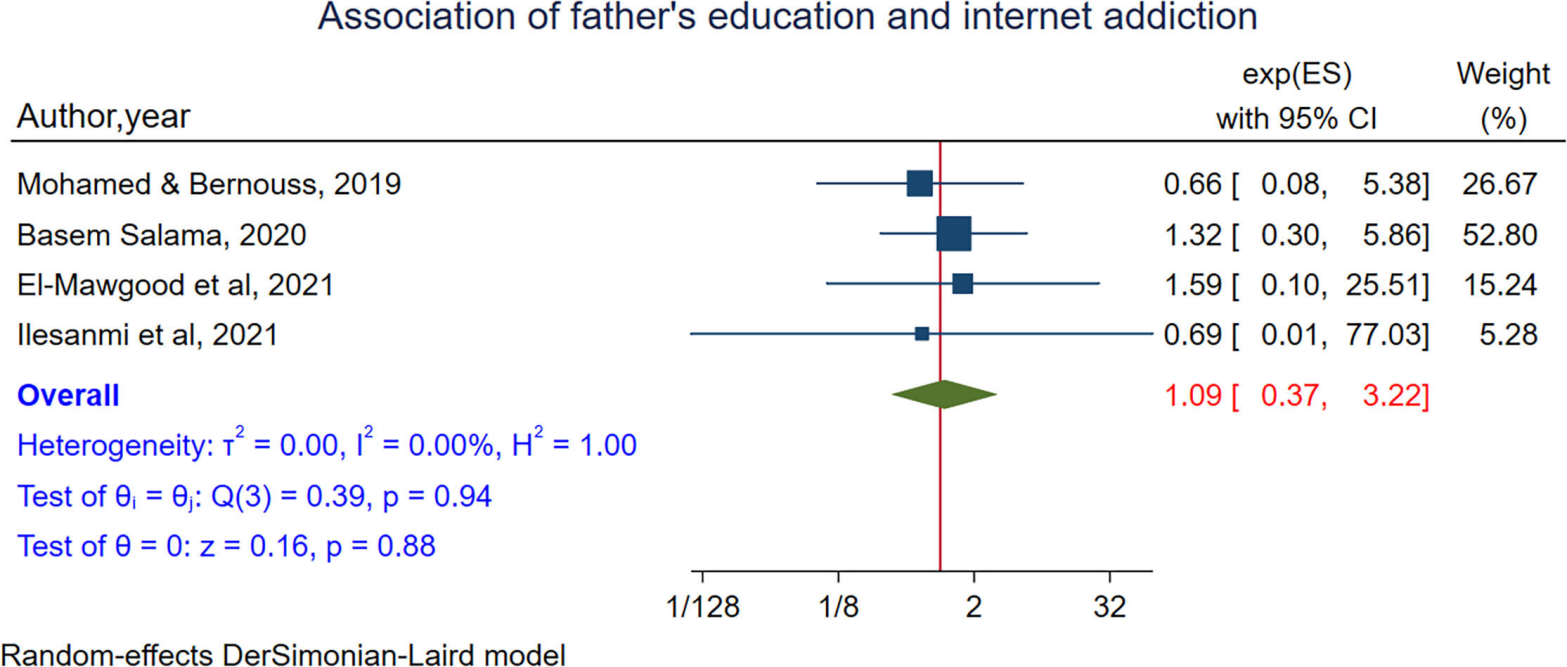
The pooled association between fathers' education and internet addiction.

Mother's and father's educational status were assessed to estimate the association between internet addiction among adolescents and the educational status of their parents. In a meta-analysis of four studies (Mohamed and Bernouss, 2020; Salama, 2020; Abd El-Mawgood et al., 2021; Ilesanmi et al., 2021), no association was found between the educational status of mothers and internet addiction among adolescents [POR = 1.18 95% CI:(0.44–3.12)]. From random-effects model, no heterogeneity was observed between studies (I^2^ = 0.00%, *P*-value = 0.82) (Figure 9). Egger's test showed no small study effect (*P*-value = 0.94). In sensitivity analysis, no single study was found to influence the estimates (Supplementary Figure 8). To assess the association between fathers' educational status and internet addiction four studies were included (Mohamed and Bernouss, 2020; Salama, 2020; Abd El-Mawgood et al., 2021; Ilesanmi et al., 2021). Again, no association was found between internet addiction among adolescents and fathers' educational status (POR = 1.09 95% CI:0.36– 3.22). From random-effects model n heterogeneity was observed between studies (I^2^ = 0.00%, *P*-value = 0.94) (Figure 10). Egger's test showed no small study effect (*P*-value = 0.84). In sensitivity analysis, no single study was found to influence the estimates (Supplementary Figure 2).

## Discussion

This review aimed to assess the prevalence of internet addiction and its associated factors among adolescents in Africa. Twenty-eight studies from 10 countries were included in the review. The overall pooled prevalence of internet addiction among adolescents was 34.53% (95% CI = 26.83–42.23). It was comparable to other meta-analyses conducted in the Gulf Countries 33% (Al-Khani et al., 2021) and Iran 31.5% (Salarvand et al., 2022). The prevalence is higher as compared to a meta-analysis conducted in China 11.3% (Li et al., 2018), South East Asia 20% (Chia et al., 2020), and with another meta-analysis conducted among health professionals 9.7% (Buneviciene and Bunevicius, 2021). The discrepancies may be due to the differences in sociodemographic background or because of the differences in the methods of assessment for internet addiction. For instance, the IAT used in this review and the review from the gulf countries is similar while for the south east Asia different internet assessment methods and cutoff points were used.

In sub-group analysis based on the economic status of the country, higher prevalence was observed in countries with upper middle income, followed by lower middle income, and lower prevalence was observed in countries of lower-income. Other studies have also shown that students with higher socioeconomic status have higher levels of internet addiction (Kayri and Günüç, 2016; Mane et al., 2018). This may be due to the reason that as the income level and GDP of countries increases the availability of smartphones and internet increases thereby increasing the prevalence of internet addiction (Bachnio et al., 2019).

In this review, male adolescents were found to have a 92% increased risk for internet addiction. There were similar findings from another review (Su et al., 2019). Different other primary studies have shown higher internet addiction among male adolescents (Ha et al., 2007; Kamal and Mosallem, 2013; Dufour et al., 2016; Liang et al., 2016; Boudabous et al., 2020; Nyaga, 2020). Gender differences can affect different addictive behaviors. Male adolescents were found to have higher addictive behaviors than females (di Nicola et al., 2017). Additionally, different studies have shown that male adolescents participate in internet pornography, gaming, and related online activities (Bruno et al., 2014; Doornwaard et al., 2016; Islam and Hossin, 2016). Which can increase addictive behaviors in male adolescents.

Again, in our review, we have found that adolescents who reside in urban areas were more likely to have internet addiction as compared with adolescents residing in rural areas. Similar findings have been reported from studies conducted elsewhere (Cao et al., 2011; Stavropoulos et al., 2013; Pawłowska et al., 2015; Karmakar, 2017). Urbanization entails increased access to internet and different determinants of addiction including psychological distress lower social interaction have been seen to be common in urban dwellers thus explaining the increase in internet addiction in urban areas (Yasuma et al., 2019; Pearlman-Avnion et al., 2020). Additionally, the pattern of internet use is different in urban areas urban students show more activities including gaming, pornography which increase the risk of addictive behaviors (Pawłowska et al., 2015).

In this review duration of internet use, more than 4 h was found to be significantly associated with internet addiction. Adolescents who stay online for more than 4 h have 2 times more risk for internet addiction. Similar findings have been reported from other studies (Tonioni et al., 2012; Beavers et al., 2015; Anusha Prabhakaran et al., 2016; Donald and Christian, 2019; Moreno-Guerrero et al., 2020). Prolonged use of internet increases the risk of addiction. Addiction and problematic use as observed from studies conducted on addiction on substances is a neuropsychological phenomenon. Neurologic pathways modulate addiction (Chou, 2005; Heilig et al., 2021). Access to substance and prolonged use of the substance has been associated with increased addictive and neurobiological behaviors (Peirce et al., 2020).

### Strength and Limitations

The strength of this study was that various databases were used to search literature, and both published and unpublished studies were included in the study. All studies included have standardized tests and the same cutoff point was used for all studies. The review has limitations, The term Internet addiction is used in this review is a general term although internet users may be addicted to a variety of activities online. Again, terminologies used to describe internet addition are different in different literature. In this review, internet addiction is used to describe a score of 50 and above on the IAT, this merges the scores for severe internet addiction and problematic internet use. Furthermore, most of the studies selected for the final analysis were conducted only in some countries of Africa, which is not the true representative of the remaining countries.

## Conclusion

Results from this review suggest that internet addiction is a major public health problem in Africa. Nearly, one-third of college and high school students in Africa are addicted to the internet. Male sex, urban residence, spending 4 h or more online were significantly associated with internet addiction. Based on the findings of this review, we recommend policymakers and all concerned bodies to give due attention to prevent and decrease internet addiction among the adolescent population.

## Data Availability Statement

The original contributions presented in the study are included in the article/Supplementary Material, further inquiries can be directed to the corresponding author.

## Author Contributions

EZ, ST, and TT involved in data conceptualization, searching critical appraisal, and statistical analysis. GA, MA, FA, GY, and TA involved study selection and quality assessment. EA, ZM, MM, TF, and MZ participated in reviewing and editing the manuscript. All authors involved in the preparation of the final manuscript and approved the final submission.

## Conflict of Interest

The authors declare that the research was conducted in the absence of any commercial or financial relationships that could be construed as a potential conflict of interest.

## Publisher's Note

All claims expressed in this article are solely those of the authors and do not necessarily represent those of their affiliated organizations, or those of the publisher, the editors and the reviewers. Any product that may be evaluated in this article, or claim that may be made by its manufacturer, is not guaranteed or endorsed by the publisher.
