# Supplementary material for: Internet Addiction and Its Associated Factors Among African High School and University Students: Systematic Review and Meta-Analysis
Source: Front Psychol. 2022 Mar 21;13:847274. doi: 10.3389/fpsyg.2022.847274 (PMC8978338; doi:10.3389/fpsyg.2022.847274)
Supplement: Supplementary file 1 [file Data_Sheet_1.PDF]

Supplemental files

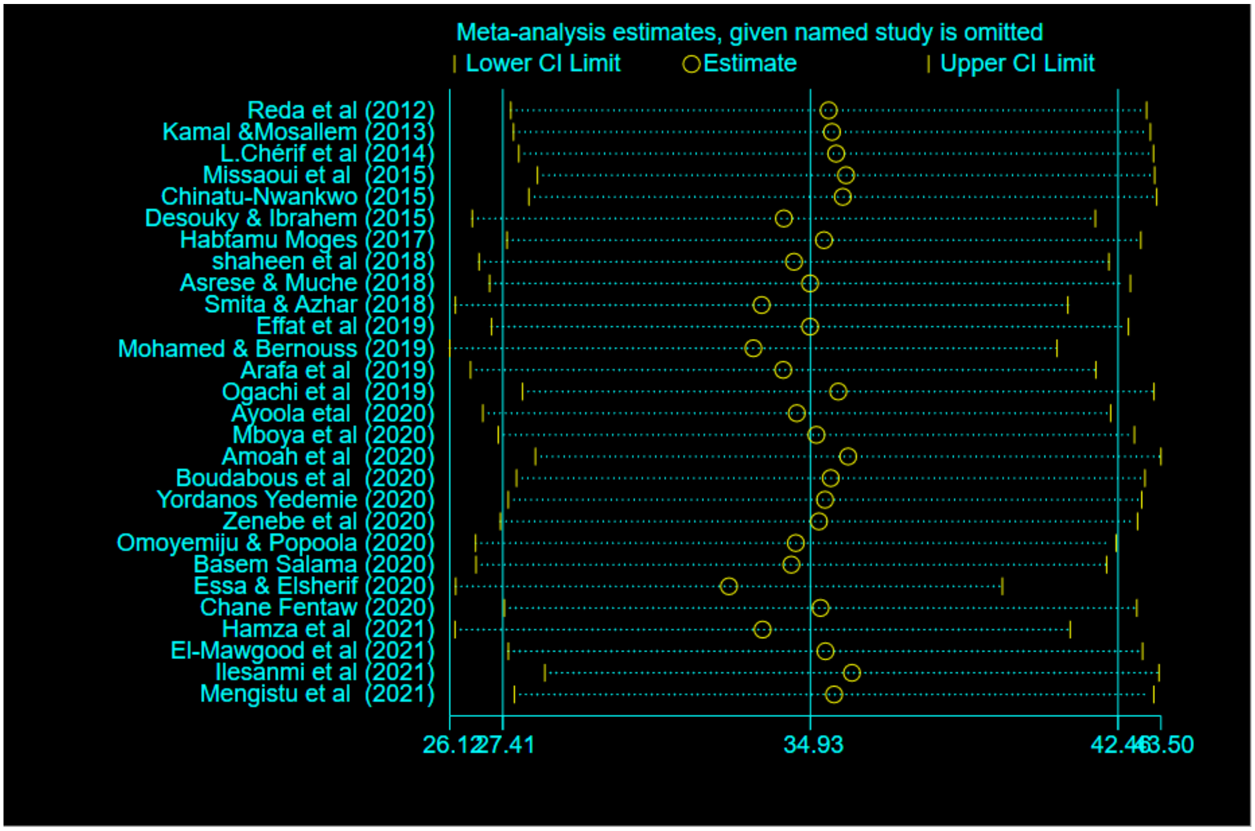

Supp fig 1. Sensitivity analysis for pooled prevalence of internet addiction among high school and university students in Africa.

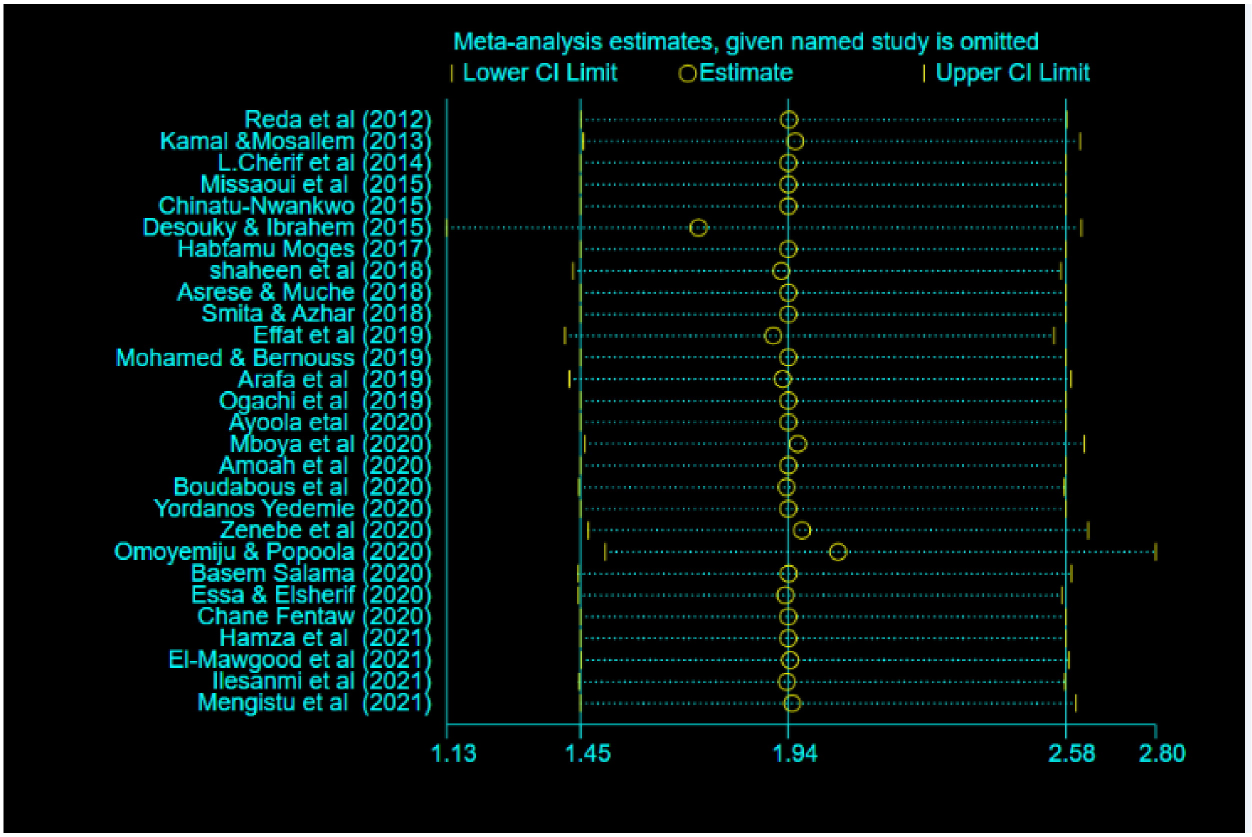

Supp fig2; sensitivity analysis for the pooled association of internet addiction and sex

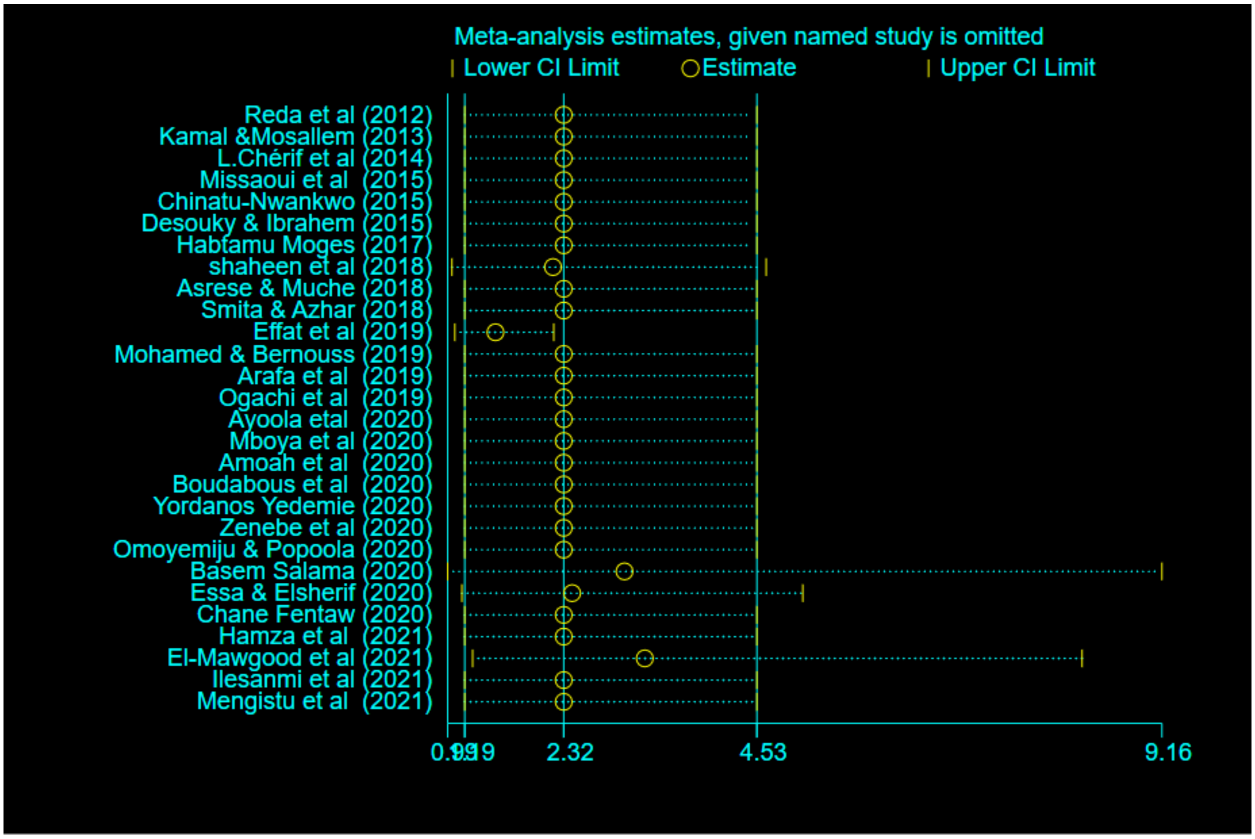

Supp fig3: sensitivity analysis for the pooled association between residence and internet addiction.

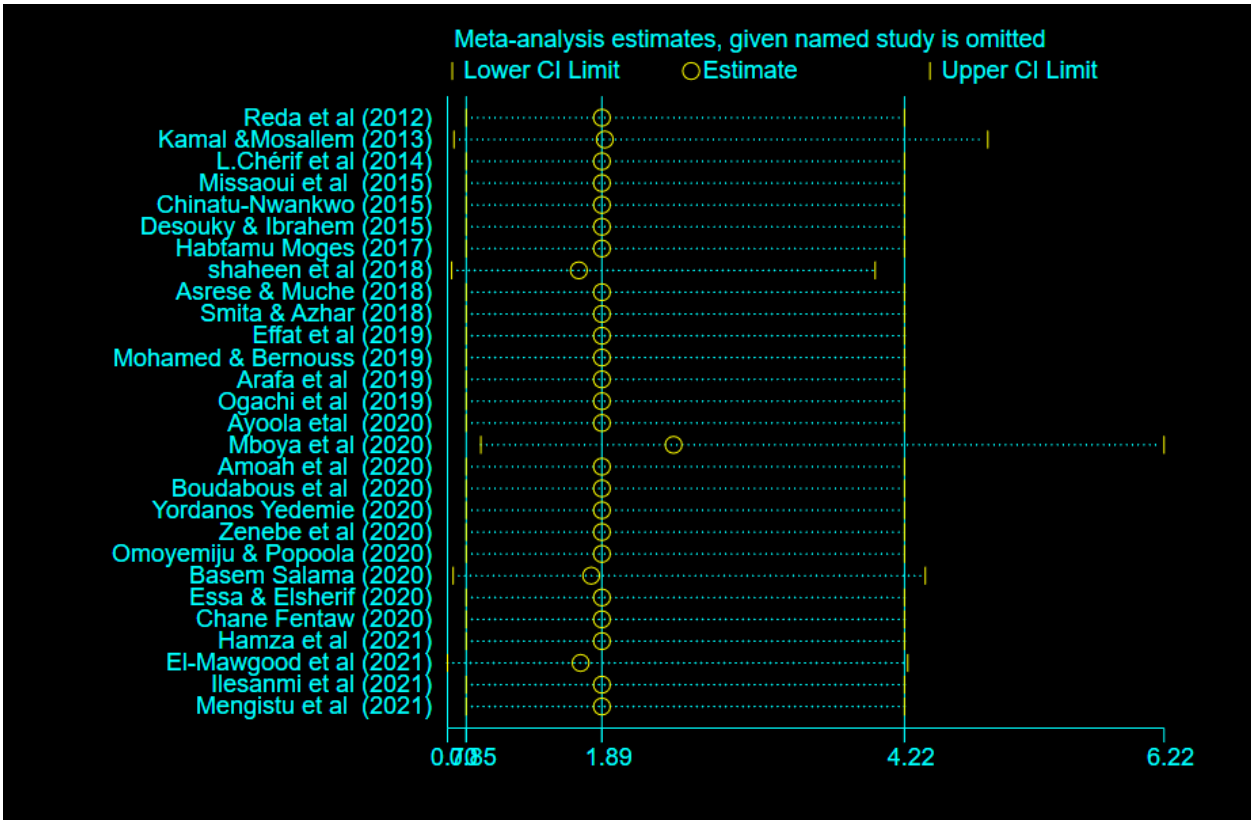

Supp fig4: sensitivity analysis for pooled association between availability of internet at home and internet addiction.

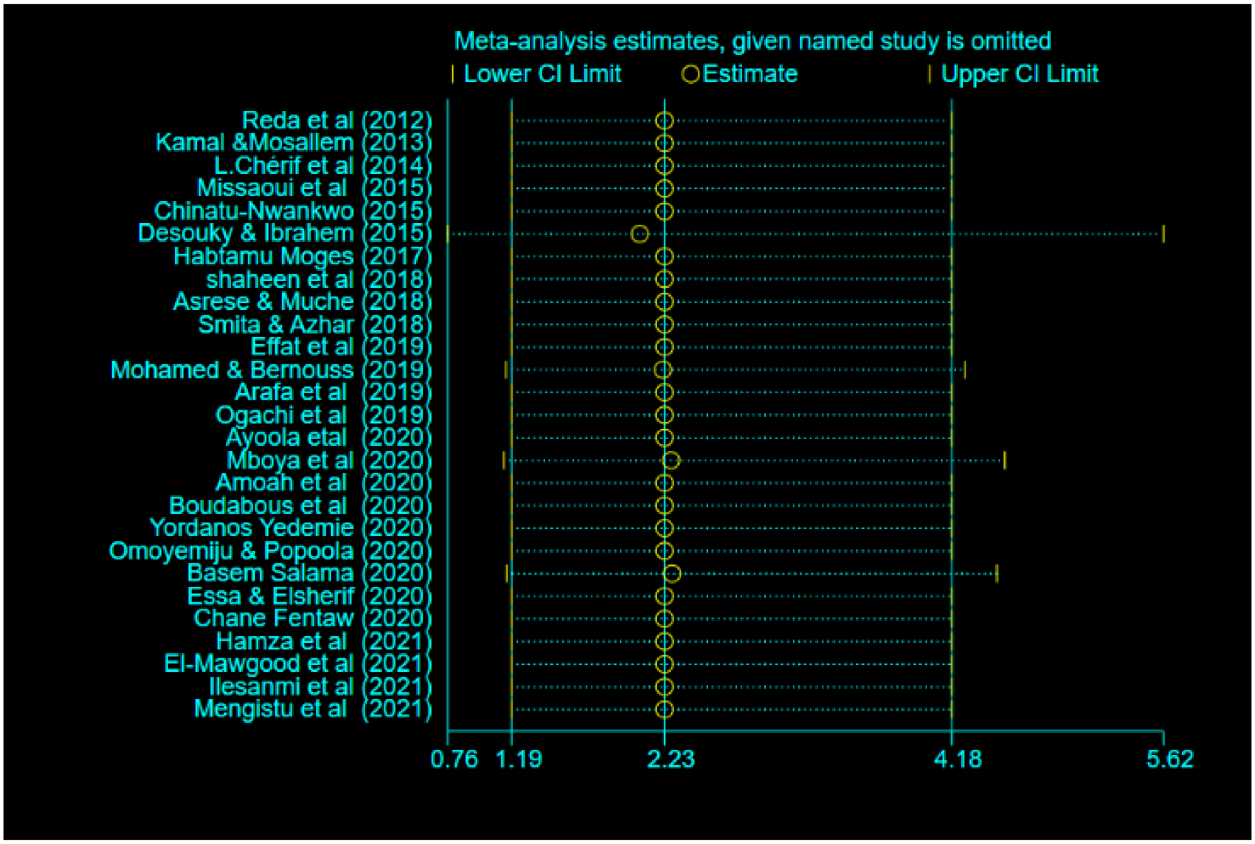

Supp fig 5: sensitivity analysis for the pooled association between duration of internet use and internet addiction.

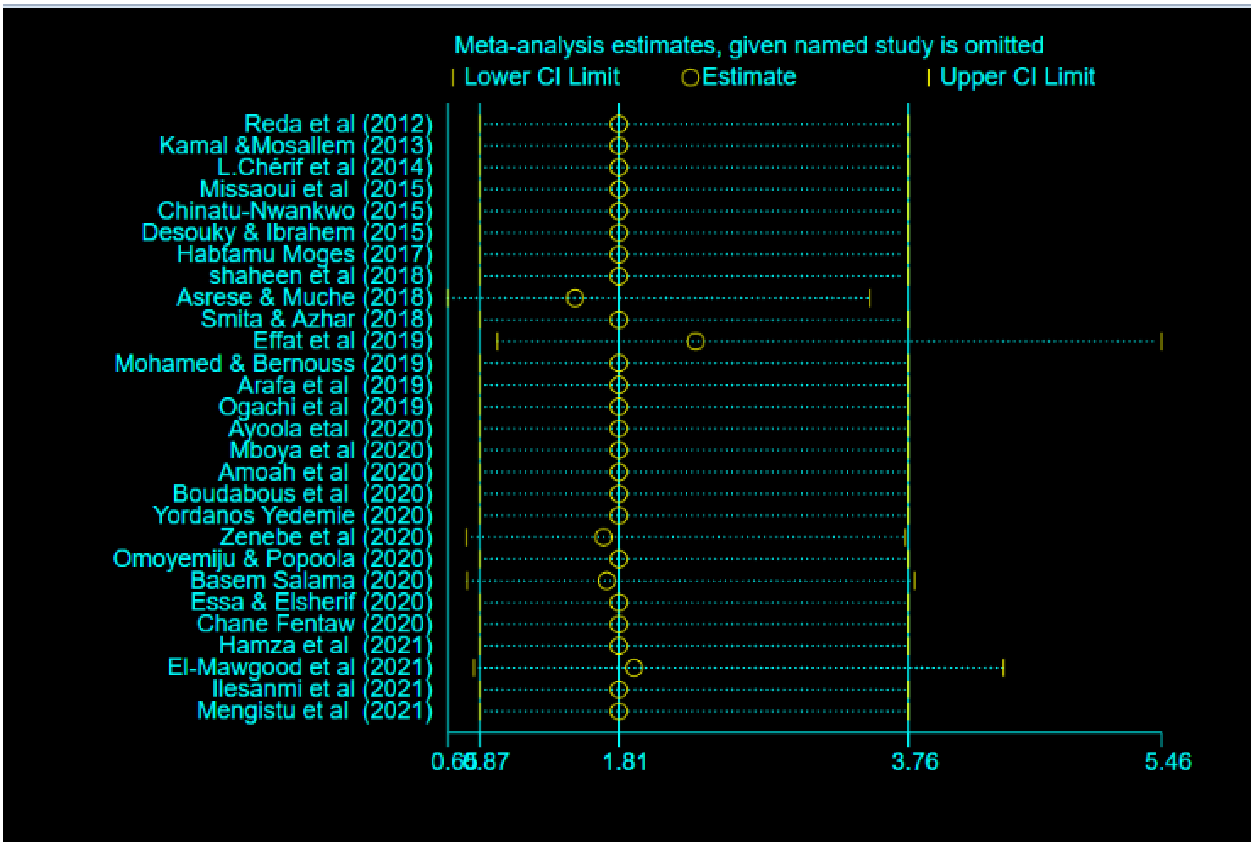

Supp fig 6: sensitivity analysis for pooled association between gaming and internet addiction

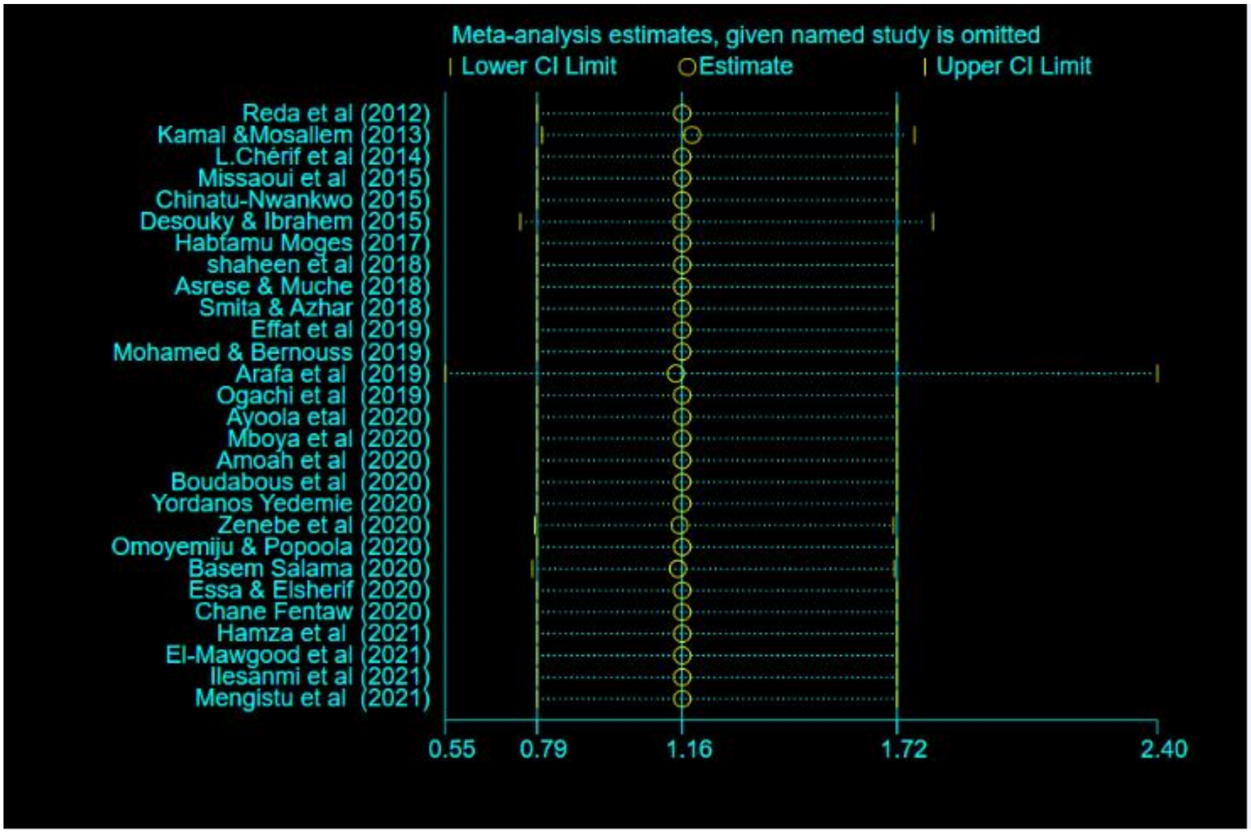

Supp fig 7: sensitivity analysis for pooled association of smoking and internet addiction.

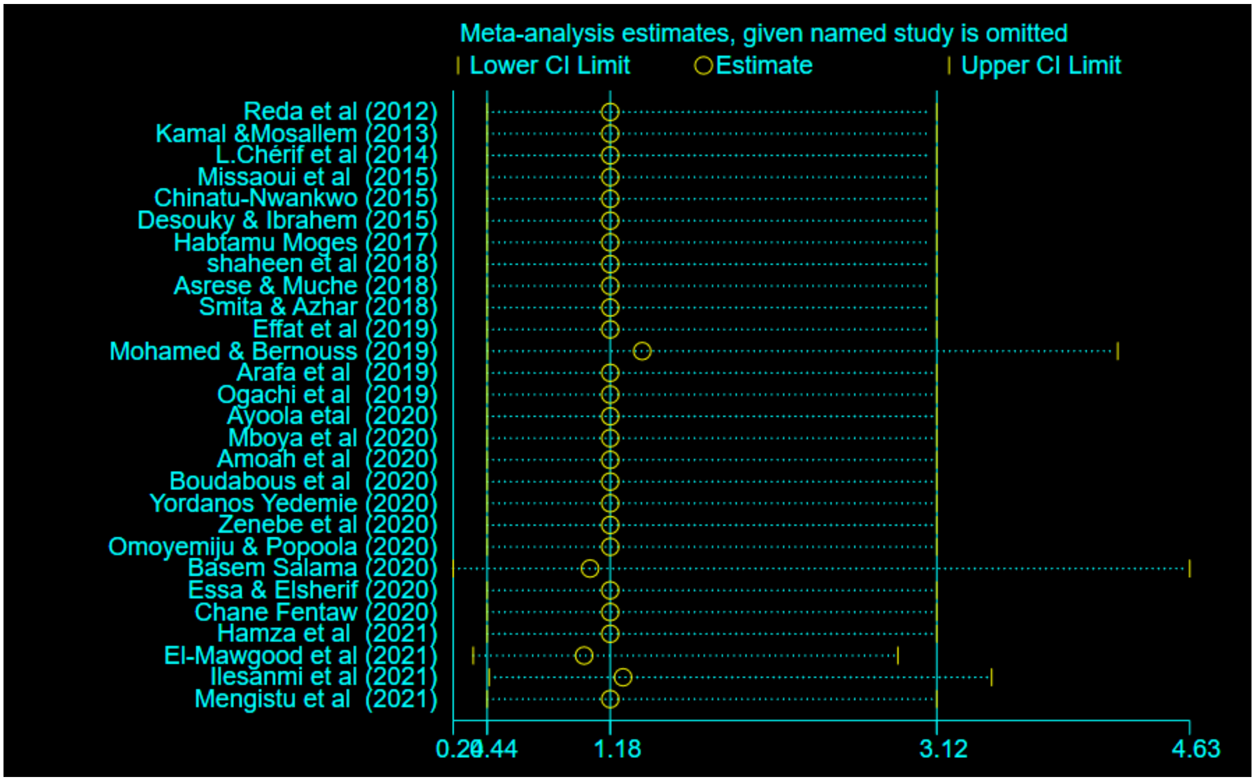

Supp fig 8; sensitivity analysis for pooled association between mother's education and internet addiction

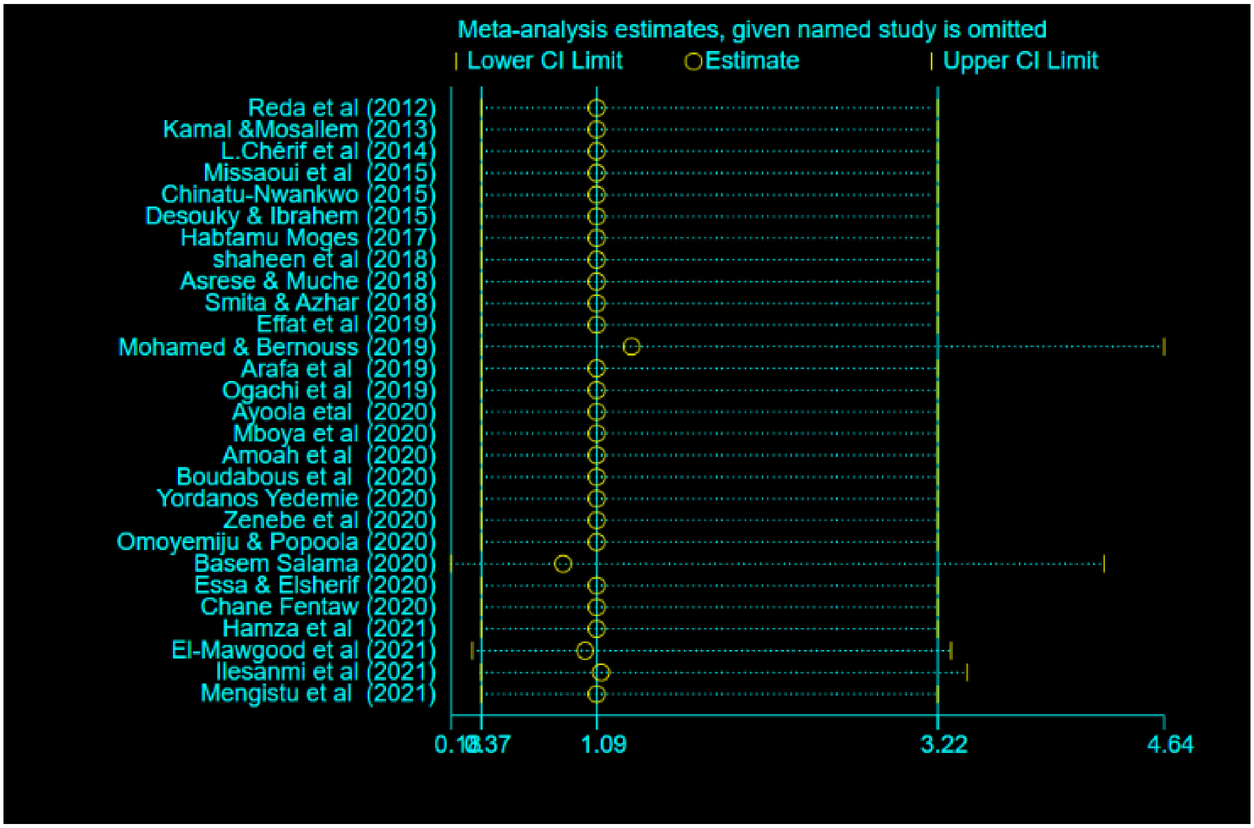

Supp fig 9: sensitivity analysis for pooled association between fathers education and internet addiction.
